# Functional study of the brassinosteroid biosynthetic genes from *Selagnella moellendorfii* in *Arabidopsis*

**DOI:** 10.1371/journal.pone.0220038

**Published:** 2019-07-25

**Authors:** Weijun Xu, Bowen Zheng, Qunwei Bai, Lei Wu, Yuping Liu, Guang Wu

**Affiliations:** 1 College of Life Sciences, Shaanxi Normal University, Xi’an, China; 2 School of Biological and Environmental Engineering, Xi’an University, Xi’an, China; Institute of Genetics and Developmental Biology Chinese Academy of Sciences, CHINA

## Abstract

Brassinosteroids (BRs) are essential hormones for plant growth and development. Enzymes DET2 and CYP90 family are responsible for BR biosynthesis in seed plants. Yet, their roles in non-seed plants are unknown. Here, we report the first functional study of *DET2* and all 4 *CYP90* genes isolated from *Selaginella moellendorfii*. *Sm89026* (*SmCPD*) belonged to a clade with *CYP90A1* (*CPD*) and *CYP90B1* (*DWF4*) while *Sm182839*, *Sm233379* and *Sm157387* formed a distinct clade with *CYP90C1* (*ROT3*) and *CYP90D1*. *SmDET2*, *SmCPD* and *Sm157387* were highly expressed in both leaves and strobili while *Sm233379* was only highly expressed in the leaves but not strobili, implying their differential functions in a tissue-specific manner in *S*. *moellendorfii*. We showed that only *SmDET2* and *SmCPD* completely rescued Arabidopsis *det2* and *cpd* mutant phenotypes, respectively, suggestive of their conserved BR biosynthetic functions. However, neither *SmCPD* nor other *CYP90* genes rescued any other *cyp90* mutants. Yet overexpression of *Sm233379* altered plant fertility and BR response, which means that *Sm233379* is not an ortholog of any *CYP90* genes in Arabidopsis but appears to have a BR function in the *S*. *moellendorfii* leaves. This function is likely turned off during the development of the strobili. Our results suggest a dramatic functional divergence of CYP90 family in the non-seed plants. While some of them are functionally similar to that of seed plants, the others may be functionally distinct from that of seed plants, shedding light for future exploration.

## Introduction

Brassinosteroids (BRs) are plant steroid hormones originally discovered in *Brassica napus* pollen [1], and later found in almost all plants examined [2]. Up to now more than 70 BRs have been identified in plants [3]. Impairing BR biosynthesis or signaling reduces plant growth and causes abnormal development, thereby limiting plant fertility and yield [4]. Therefore, BRs play a broad role in plant growth and development.

**Fig 1:**
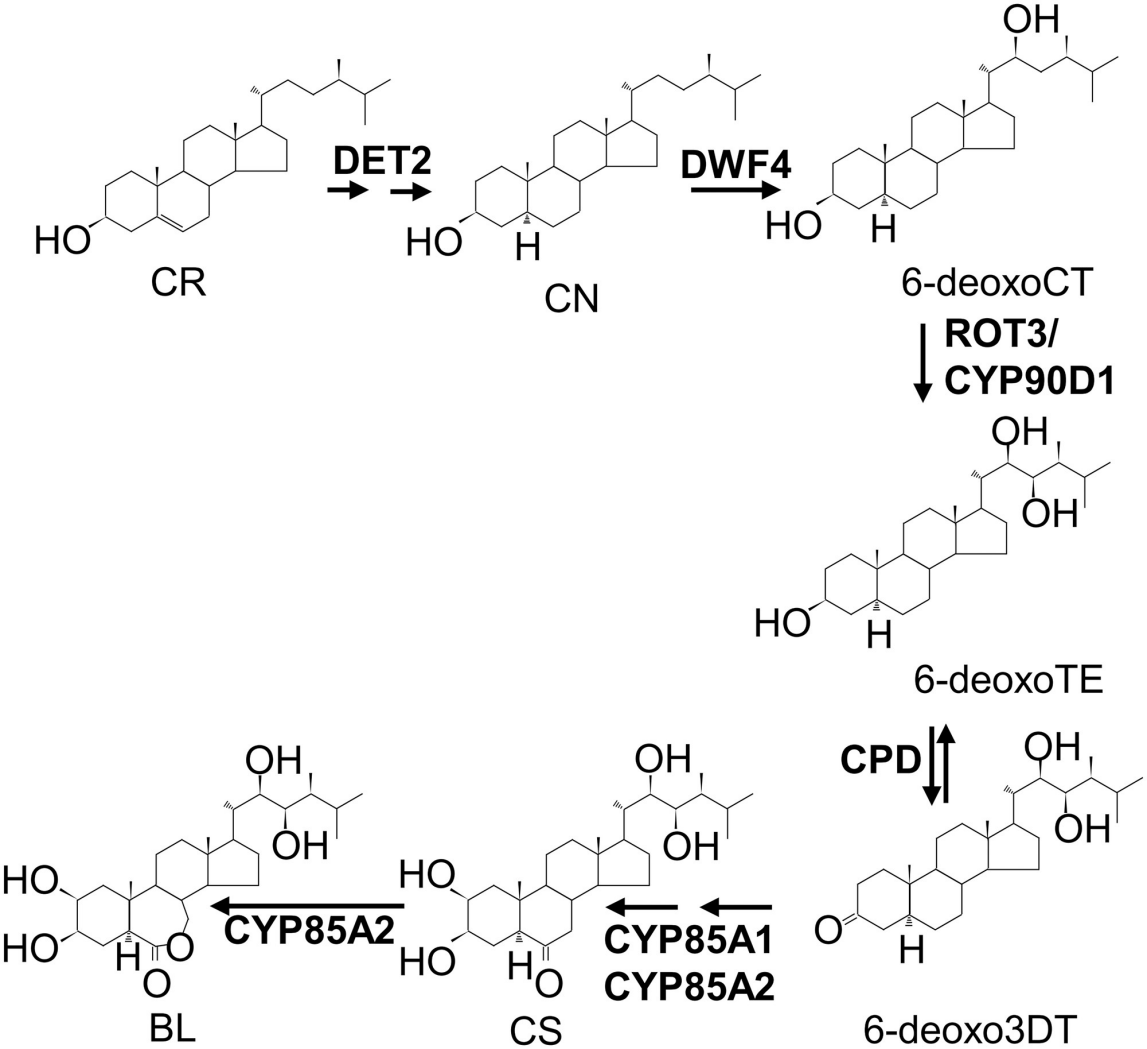
A simplified CN-dependent schematic process of brassinosteroid biosynthesis.

At present, it is known that brassinolide (BL), the most active BR, is converted from castasterone (CS). CS is synthesized from campestanol (CN) through either the early C-6 oxidation and/or the late C-6 oxidation pathway(s) [5]. The first reaction toward the BL is the conversion of campaesterol (CR) into CN, which is then converted into 6-deoxocathasterone (6-deoxoCT). This process has been demonstrated in cultured cells of *Catharanthus rosenus*. Through feeding test, 6-deoxoteasterone (6-deoxoTE) is detected as a major metabolite from 6-deoxoCT. At the end, 6-deoxoTE is finally converted into CS, BL, in that sequence (Fig 1). BR biosynthesis is achieved from CR to BL not by single but by parallel and highly branched pathways [6,7].

The Lycophytes are the earliest group of vascular plants entering onto the earth, playing a critical role in the evolution of plants. In view of this fact, the *Selaginella moellendorfii*, a species of Lycophytes, was taken as the material to study the function of BR biosynthesis genes. As reported previously, there are homologous genes involved in BR biosynthesis in *Selaginella*, which include *DET2*, *Sm89026*, *Sm182839*, *Sm233379* and *Sm157387* [8]. However, whether they have biological functions has not been explored.

In this work, we demonstrate that *SmDET2* can recover the *det2* mutant and *Sm89026* can rescue the *cpd* mutant. This implies that *SmDET2* and *Sm89026* are likely functional in *S*. *moellendorfii*. However, *Sm182839*, *Sm233379* and *Sm157387* clustered in the same clade with *ROT3* cannot rescue *cpd*, *rot3* or *dwf4*, but overexpression of *Sm233379* does enhance a BR function and impact plant fertility. Additionally, we do not find *ROT3* homologous gene in the monocots. Taken together, these results lead us to propose that there is similar BR biosynthesis pathway from *S*. *moellendorfii* to *Arabidopsis thaliana*, with *SmDET2* and *Sm89026* (*SmCPD*) playing a key role in *Selaginella*. Additionally, *Sm233379* is involved in BR biosynthesis distinct from that of *AtROT3* in the same clade. Yet, only future exploration can resolve the exact function of *Sm233379*, *Sm182839* and *Sm157387* in *S*. *moellendorfii*.

## Materials and methods

### Phylogenetic and schematic analysis

The amino acid sequences of the enzymes involved in the BR biosynthesis were extracted from NCBI (https://www.ncbi.nlm.nih.gov/). Full-length protein sequences were aligned with ClustalX2. Phylogenetic trees were generated using the Neighbor-joining method with MEGA7 software.

### Plant material

*Arabidopsis thaliana* ecotype Columbia (Col-0) was used as the wild-type (WT) control. The BR-related mutants, *cpd*, *rot3* and *dwf4*, transgenic *SmCYP90* (*Sm89026*, *Sm182839*, *Sm233379*, *Sm157387*) lines, and overexpression lines (*Sm89026-OX*, *Sm182839-OX*, *Sm233379-OX* and *Sm157387-OX*) were all in the Col-0 background. Seeds were imbibed for 4 days at 4°C, then transplanted onto the soil. Plants were grown at 22°C with 70% humidity under a 16-h light (~120μmol.m^-2^.s^-1^)/8-h dark cycle.

### Vector constructs and transgenic lines

Full-length gDNAs of *DET2* and *CYP90* genes without a stop codon were amplified by PCR using gene-specific primers (S1 Table) with 30 cycles. The amplified DNAs were then inserted into the plant expression vector (p35S-CHF3-GFP) individually to generate *p35S*:*AtDET2-GFP*, *p35S*:*AtCPD-GFP*, *p35S*:*AtROT3-GFP*, *p35S*:*AtDWF4-GFP*, *p35S*:*SmDET2-GFP*, *p35S*: *Sm89026-GFP*, *p35S*:*Sm182839-GFP*, *p35S*: *Sm233379-GFP*, and *p35S*: *Sm157387-GFP*. These constructs were then transferred into plants via Agrobacterium (GV3101)-mediated transformation using the floral dip method [9]. The transformants were screened on 1/2 MS with 50 μg/mL kanamycin.

### Hypocotyl growth analysis

Seeds were surface-sterilized and placed on half strength MS plates with 0.8% (w/v) agar, 1% (w/v) sucrose, with or without BRZ (brassinazole) with a concentration of 5 μM. The plates were cold treated at 4°C for two days to ensure uniform germination. Seeds were considered to begin germination after the plates were kept at 22°C for 24 h. Five DAG (days after germination) in the dark, seedlings were put on a transparent film and scanned to acquire images, which were then used to measure the hypocotyl length with ImageJ (http://rsb.info.nih.gov/ij/). For statistical comparisons, LSD (least significant difference) test was performed (p<0.01).

### Light microscopy observation

Fresh buds approximately containing stage-8 anthers were fixed, dehydrated as described by Zhang et al., then stained with Alexander’s staining solution [9].

### Semi-quantitative RT-PCR

Total RNAs were extracted using a HiPure Plant RNA Mini Kit (Magen, R4151-02, China) according to the manufacturer’s protocol. First–strand cDNA was synthesized from 1μg of total RNA using M-MLV First Strand cDNA Synthesis Kit (Omega, TQ2501-02, Norcross, GA, USA). Semi-quantitative RT-PCR (PCR for genes of *Arabidopsis thaliana* ran 30 cycles, except for *BAS1* that ran 32 cycles. PCR for genes of *S*. *moellendorfii* ran 31 cycles) analyses were performed using specific primers to study the expression levels. The primers used were listed in the S2 Table.

## Results

### *DET2* has a conserved function

**Fig 2:**
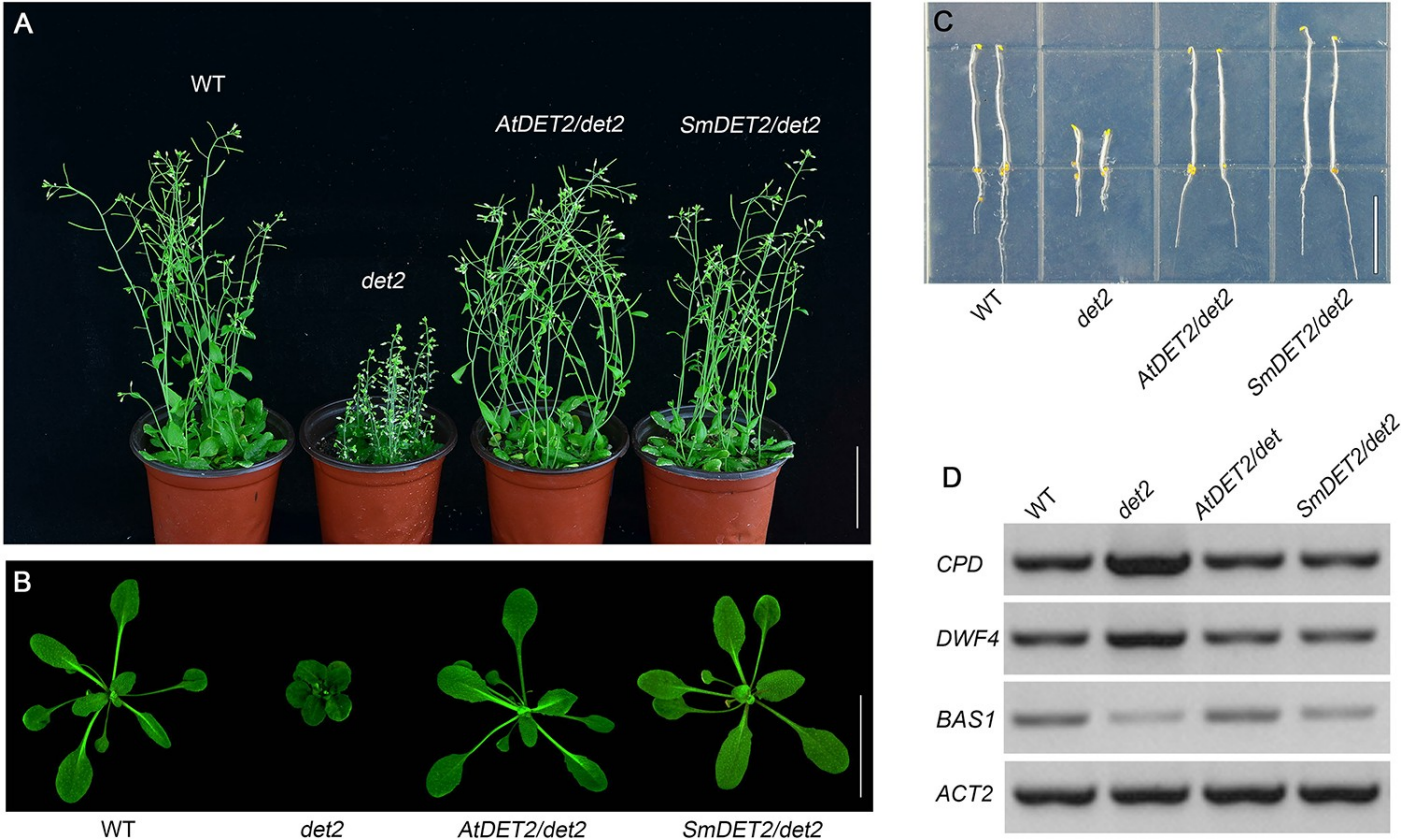
*SmDET2* completely rescued the phenotypes of Arabidopsis *det2* mutants. (A) Phenotypes of WT, *det2*, *AtDET2/det2* and *SmDET2/det2* 30 DAG (days after germination). Scale bar, 5 cm. (B) Phenotypes of 6-week plants grown under a long-day condition (16/8 h, light/dark). Scale bar, 5 cm. (C) hypocotyls 5-DAG dark-grown seedlings in 1/2 MS medium. Scale bar, 1 cm. (D) RNAs were prepared from wild-type (WT), *det2* mutant (*det2*), and transgenic seedlings in *det2* background grown in glass jars under white light for 10 days, semi-quantitative RT-PCR analysis of the transcripts of *CDP*, *DWF4* and *BAS1*. To control equal loading of RNA samples, *(At)ACT2* gene served as a control.

Our results showed that, after 5 days of growth in total darkness, the *det2* mutants were short with thick hypocotyls, and opened and expanded cotyledons (Fig 2C), consistent with previous reports [10]. In order to determine the function of *SmDET2*, we expressed it under the control of CaMV *35S* promoter. We found that it completely rescued the *det2* mutant phenotypes. The transgenic plants showed normal adult phenotypes (Fig 2A), normal young plants (Fig 2B) and normal seedlings in the dark (Fig 2C). BRs activate the activities of the BES1 family transcription factors that downregulate the expression of BR biosynthetic genes, such as *CPD* and *DWF4*, and upregulate BR metabolic genes, such as *BAS1* [11]. Thus, this phenomenon has been used as a reliable marker for the presence of BRs or their signaling [11]. Therefore, to more accurate estimate the role of *SmDET2* in above transgenic plants, semi-quantitative RT-PCR with the total RNAs prepared from these seedlings was performed. We confirmed that *SmDET2* rescued the *det2* mutant phenotypes at a level of wild type plants (Fig 2D), which means that *SmDET2* and *DET2* have a conserved function. It is worth mentioning that the phenotypes of the expression of *SmDET2* and *AtDET2* in the *det2* mutants were completely indistinguishable (Fig 2D). We thus conclude that SmDET2 and AtDET2 have very similar if not the same function.

### Phylogenetic analysis of CYP90 enzymes involved in BRs biosynthesis

**Fig 3:**
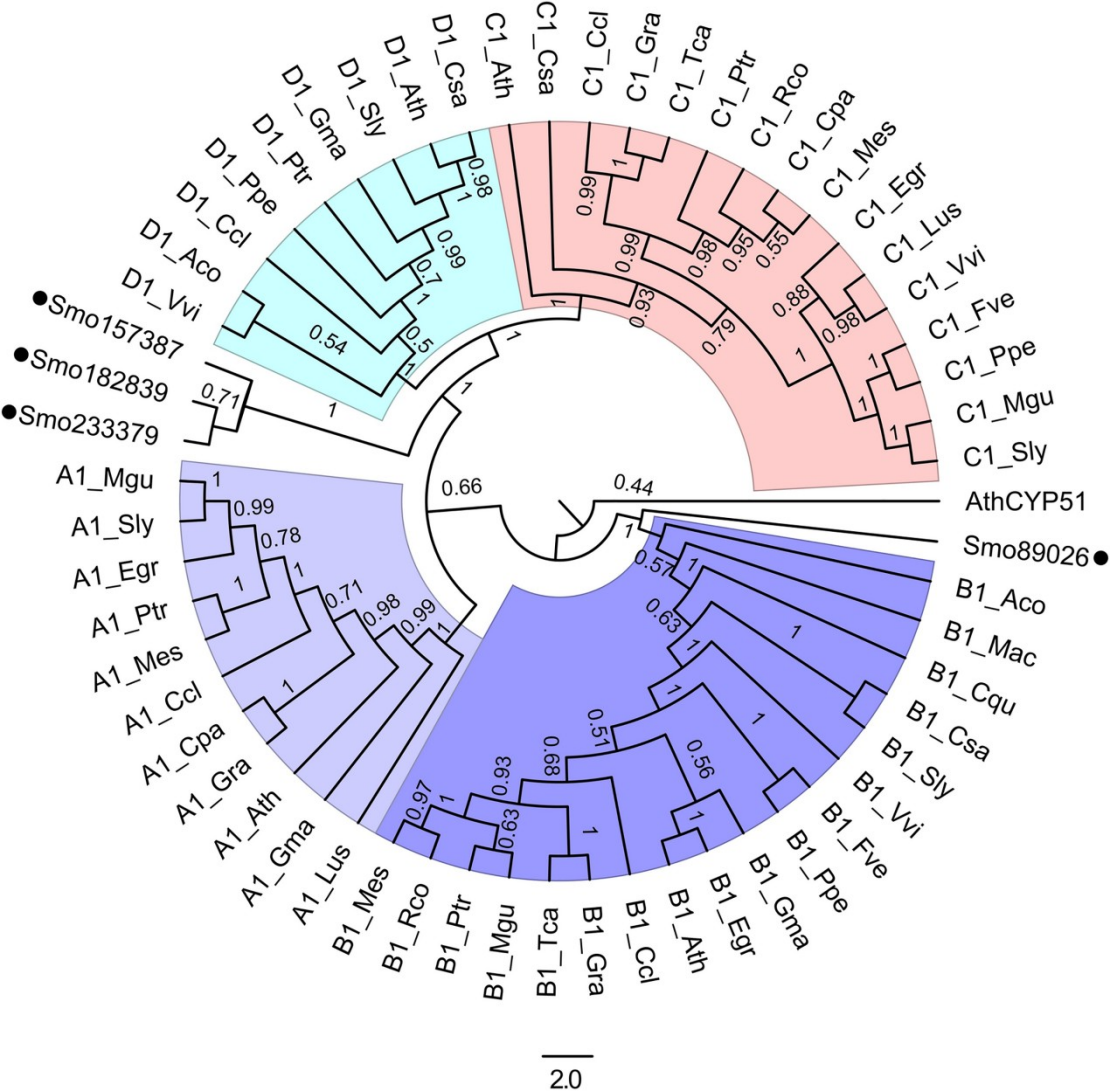
NL tree of CYP90 family enzymes. The phylogenic relationship of CYP90 enzymes in various plants and *S*. *moellendorfii* is shown. Full-length protein sequences were aligned with Clustal X2. A1, B1, C1 and D1 are short for CYP90A1, CYP90B1, CYP90C1 and CYP90D1, respectively. AthCYP51 served as an out-group. Bootstrap decimals were indicated at the branch points. The full names of the species used were showed in S3 Table.

Besides DET2, CPD, DWF4, ROT3 and CYP90D1 are also the critical enzymes that catalyze the important reactions in the later steps of BRs biosynthesis (Fig 1). Sm89026 was previously named SmCPD [8]. However, protein sequence comparison and our phylogenetic analysis revealed that Sm89026 was in a clade with DWF4 sister with CPD while Sm182839, Sm233379 and Sm157387 formed a distinct clade with ROT3 and CYP90D1 (Fig 3) [8]. These results suggest that either CPD or DWF4 is lost in *S*. *moellendorfii* (Fig 3). Thus, there is a need to determine whether Sm89026 functions as a CPD or a DWF4.

### *CYP90* genes are differentially expressed in *Selaginella moellendorfii*

**Fig 4:**
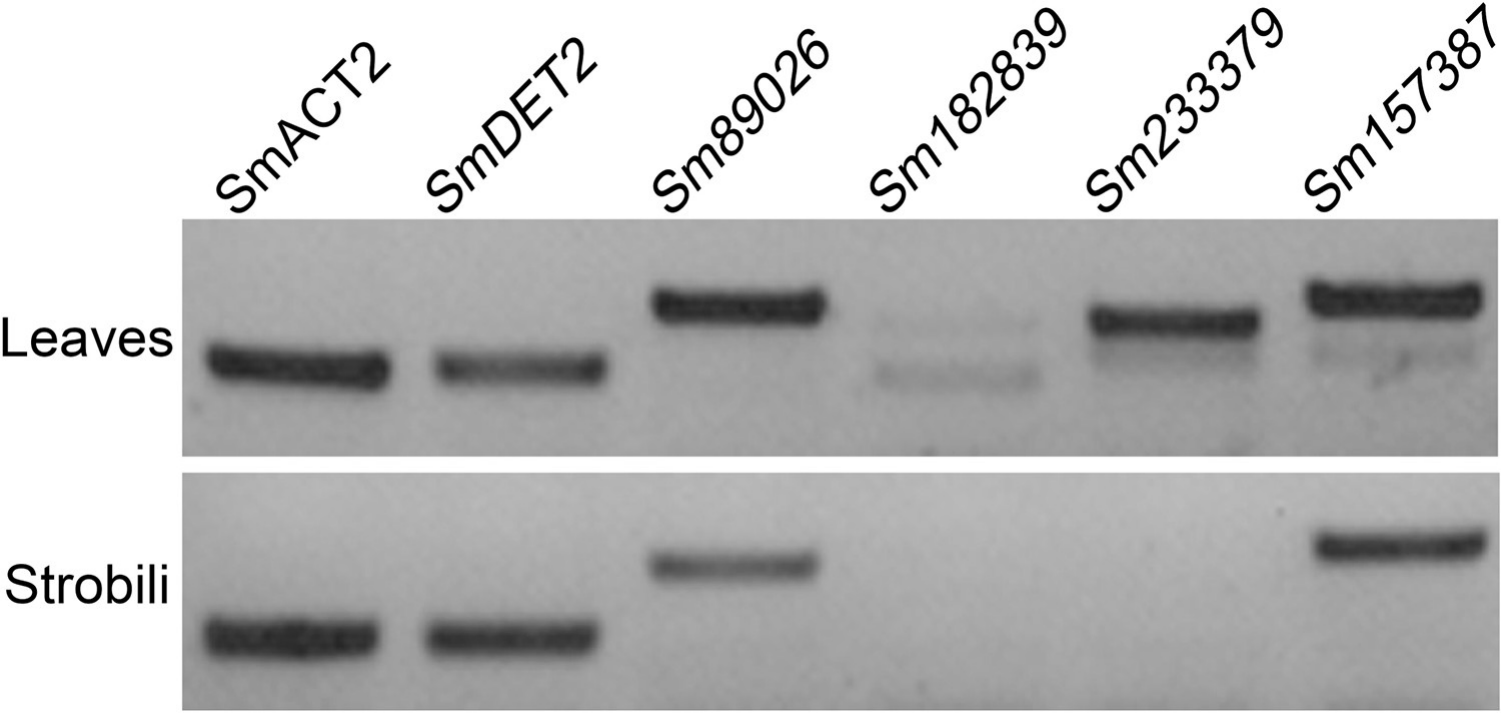
Differential expression of *SmDET2* and *SmCYP90* genes. *SmACT2* served as a control.

Gene expression patterns are major parameters of how genes function. Using semi-quantitative RT-PCR, we analyzed the expression level of *CYP90* genes in a vegetative organ, leaf, and a reproductive organ, strobili, of *S*. *moellendorfii*. We demonstrated that both *Sm89026 (SmCPD*) and *Sm157387* were highly expressed in both organs while *Sm233379* was only significantly expressed in the leaves but not strobili. However, *Sm182839* was under-expressed in both tissues (Fig 4). These results suggest an expression divergence in *SmCYP90* genes. Together with their sequence divergence, this may indicate that *SmCYP90* genes have distinct functions from each other.

### *CYP90* genes involved in BR biosynthesis in *Selaginella moellendorfii*

**Fig 5:**
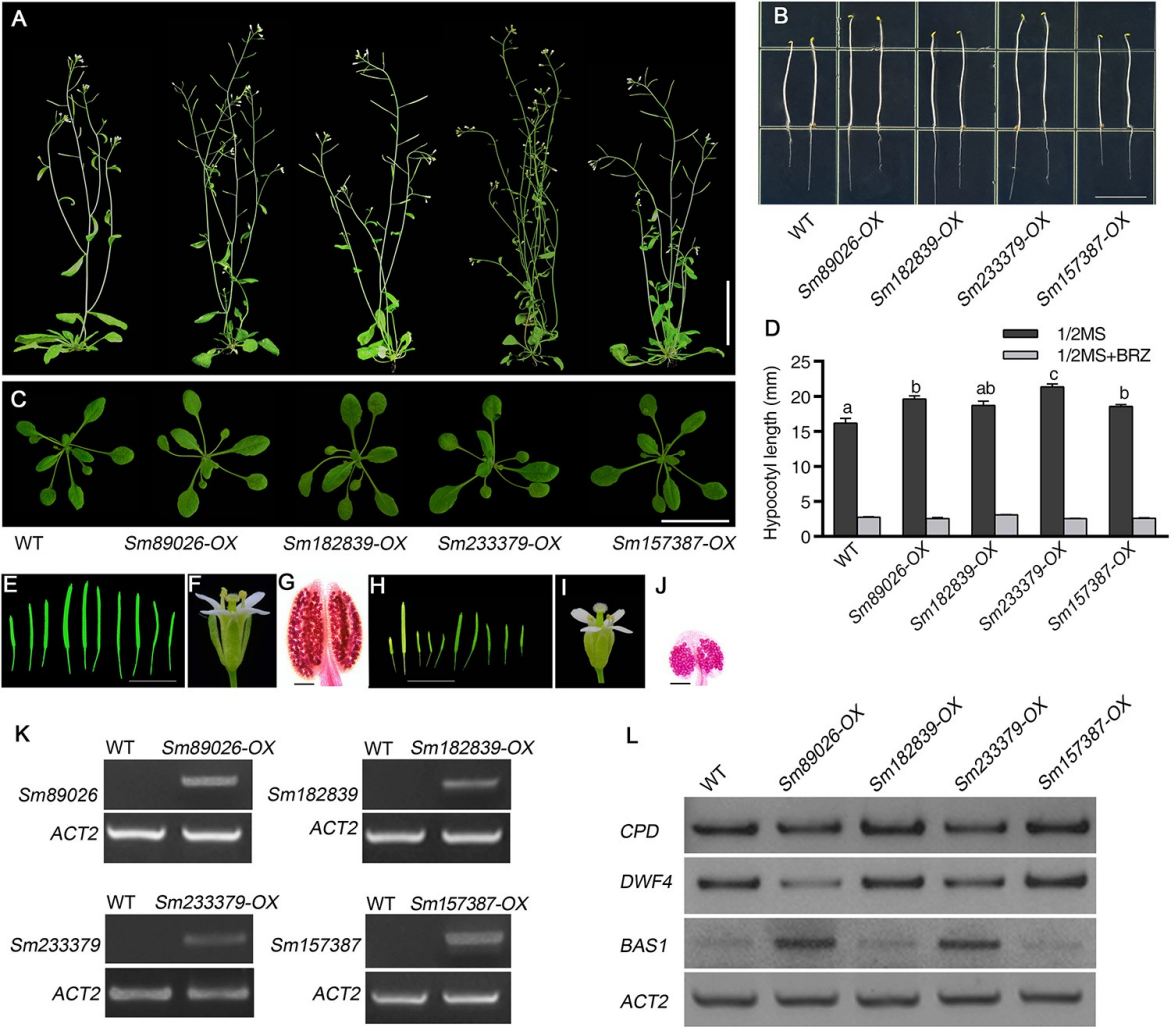
Phenotypes of the WT (wild-type) and overexpression of *SmCYP90* genes. (A) Morphology of WT and overexpression of *SmCYP90* genes mature plants grown 6-week after germination under a long-day condition (16/8 h, light/dark). Scale bar, 5 cm. (B) Morphology of the WT and lines with the overexpression of *SmCYP90* seedlings grown on 1/2 MS medium 5 DAG in dark. Scale bar, 1.0 cm. (C) Morphology of WT and overexpression of *SmCYP90* seedlings grown in soil 30 DAG under a long-day condition (16/8 h, light/dark). Scale bar, 3 cm. (D) Analysis of the length of hypocotyls in the seedlings of WT and lines with the overexpression of *SmCYP90*. Seedlings were grown on 1/2 MS medium in dark for 5 days. Values represented the mean of 30 measurements ± SD. Letters above each bar indicated a significant difference compared to the mock treatment. (E) Siliques of the WT. (F) A mature flower of the WT. Scale bar, 1 cm. (G) An anther of the WT. Scale bar, 50 μm. (H) The fertility of *Sm233379-OX* was largely reduced, which was indicated by the fact that most of siliques were completely or partially lacked of seeds. Scale bar, 1 cm. (I) The length of the stamens in *Sm233379-OX* was shorter than the stigma. (J) Anther staining examination revealed a reduction of viable pollen grains in *Sm233379-OX* anthers, compared to the WT. Scale bar, 50 μm. (K) Semi-quantitative RT-PCR analysis of the expression of *SmCYP90* genes in 10-day-old seedlings of the transgenic lines was shown. *ACT2* served as an internal control. (L) RNAs were prepared from seedlings grown in glass jars under white light for 10 days. Semi-quantative RT-PCRs were shown for the WT and transgenic lines of *SmCYP90* genes (*Sm89026*, *Sm182839*, *Sm233379* and *Sm157387*). *ACT2* served as an internal control. Our analysis indicated that the *CPD* and *DWF4* genes were down-regulated while *BAS1* was up-regulated.

To ask whether *SmCYP90* genes indeed encode enzymes involved in BR biosynthesis in *S*. *moellendorfii*, we cloned *SmCYP9*0 genes under the control of *35S* promoter and expressed them in wild type (WT) *Arabidopsis* plants. Most of these transgenic plants more or less resembled BR overproducing plants (Fig 5A–5D and 5L and S1 Fig) [12–13]. They showed longer petioles, larger rosette diameters, less rosette leaves and slender shoots (Fig 5A and 5C and S2 Fig). We further observed the elongated hypocotyls in all these over-expressing lines (Fig 5B and 5D) [14]. Yet, they were all sensitive to BRZ (brassinozole) that specifically inhibits BR biosynthesis (Fig 5D and S3 Fig). Surprisingly, using upregulation of *CPD* and *DWF4* and downregulation of *BAS1* as the indicators of BR overproducing markers, only the transgenic plants of *Sm89026 (SmCPD*) and *Sm233379* had undoubted BR overproducing phenotypes (Fig 5L). Nevertheless, an enhanced expression of *BAS1* in *Sm89026-OX* and *Sm233379-OX* plants suggests a role of *Sm89026* and *Sm233379* in positive regulation of BR biosynthesis.

Intriguingly, the fertility of overexpression *Sm233379* was dramatically reduced, compared to the WT (Fig 5A and 5H–5J, S4 Fig). Reduced fertility can be induced by many reasons, such as abnormal tapetum [15], impaired pollen tube-stigma interaction [16], failure of pollen tube integrity and sperm release [17], etc. We found that *Sm233379-OX* plants had little male fertility, and produced shorter siliques than WT plants. To further find the reason for the reduced fertility, we dissected the flower of *Sm233379-OX*, and found that the majority of transgenic plants with *Sm233379-OX* had fewer pollen grains with small size. As *Sm233379* was only expressed in vegetative tissues of *S*. *moellendorfii* (Figs 4 and 5A), thus the fertility phenotypes could be a result of spatiotemporal mis-localization of certain BRs in the reproductive organs of the transgenic plants [18]. Phenotypic differences in the transgenic lines of the overexpression of *CYP90* genes suggest that the BR signaling in these plants might be altered. Therefore, we analyzed transcript levels of *(At)CPD*, *(At)DWF4* and *(At)BAS1* using semi-quantitative RT-PCRs in these transgenic plants and found that the transcript levels of all three genes were altered in *SmCYP90-OX* seedlings as compared with those of WT (Fig 5K and 5L).

### *Sm89026* is a functional equivalent of *CPD* in *Selaginella moellendorfii*

**Fig 6:**
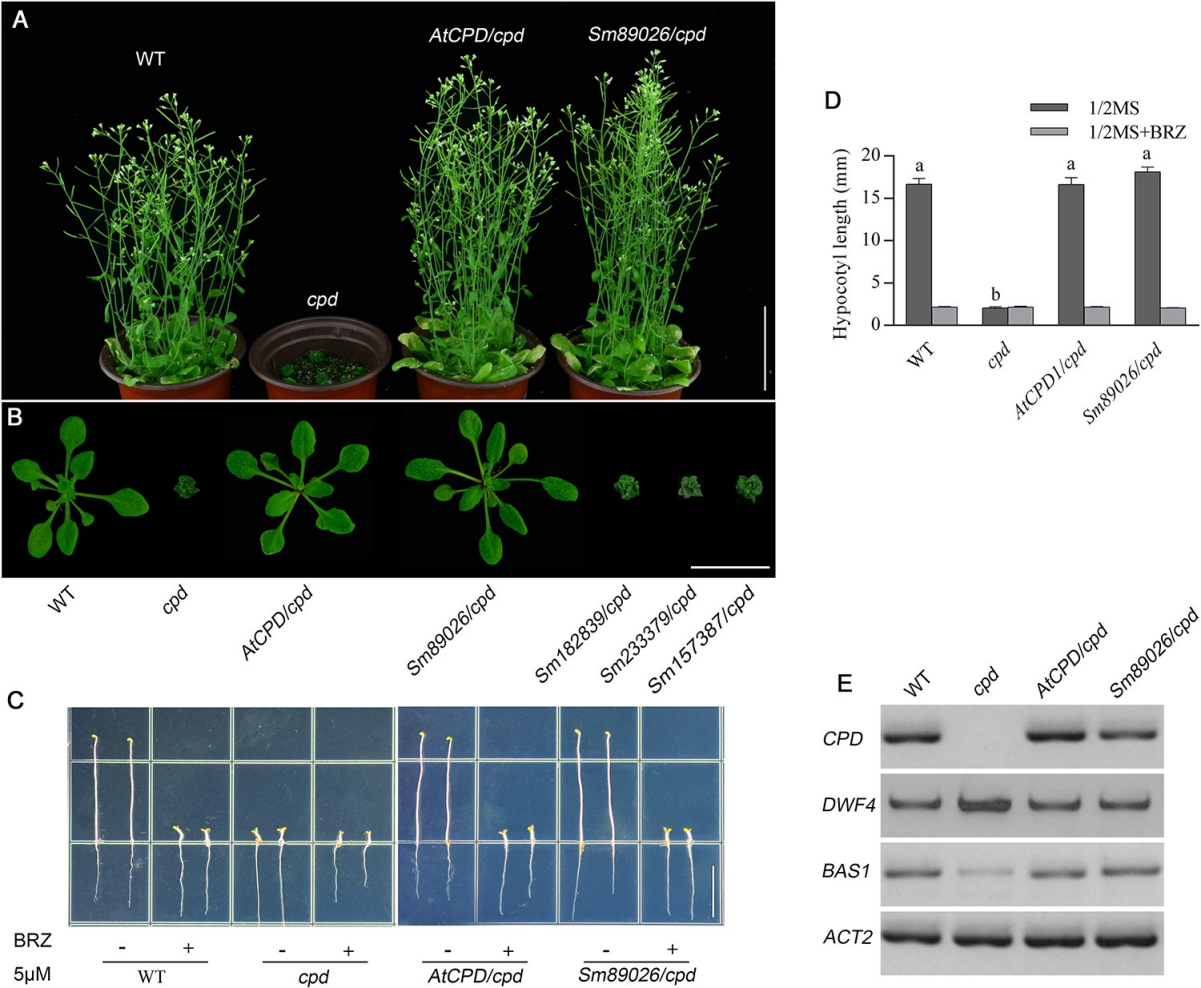
Comparison among WT, *cpd*, *AtCPD1/cpd* and *SmCYP90*/*cpd* plants. (A) Pictures from WT, *cpd*, *AtCPD/cpd* and *Sm89026/cpd* plants grown 6-week after germination. Scale bar, 5 cm. (B) Morphology of WT, *cpd*, *AtCPD/cpd* and *SmCYP90* genes and seedlings grown 30 DAG. Scale bar, 3 cm. (C) Morphology of WT, *cpd*, *AtCPD/cpd* and *Sm89026/cpd* seedlings grown five DAG in dark on 1/2 MS medium with or without BRZ (brassinazole). Scale bar, 1.0 cm. (D) Analysis of the hypocotyl length of 5-DAG dark-grown WT, *cpd*, *AtCPD/cpd* and *Sm89026/cpd*. Values represented the mean of 30 measurements ± SD. Letters above each bar indicated a significant difference compared with the mock treatment. (E) Semi-quantitative PCR analysis expression of *CPD*, *DWF4* and *BAS1* in the 10-day-old WT, *cpd*, *AtCPD/cpd* and *Sm89026/cpd*. The *AtACT2* gene served as a control.

Among the transgenic lines of *SmCYP90* family, only *Sm89026/cpd*, like *AtCPD/cpd*, had normal phenotypes as the WT, which led to the suggestion that *Sm89026* can completely rescue the phenotypes of *cpd* (Fig 6A–6D), indicating that *Sm89026* is functionally equivalent to the *Arabidopsis CPD*. To investigate if the morphological evidence for the BR biosynthesis was consistent with the indication at the molecular level, the expression of *CPD*, *DWF4* and *BAS1* was analyzed using semi-quantitative PCRs in WT, *cpd* and *AtCPD/cpd* and *Sm89026/cpd* seedlings. We found that the expression of *BAS1* was significantly decreased in *Sm89026/cpd* plants, whereas the expression of *CPD* and *DWF4* was slightly reduced (Fig 6E), supporting that *Sm89026* functions as an *Arabidopsis CPD*.

### No functional equivalent of *DWF4* and *ROT3* found in *SmCYP90*

**Fig 7:**
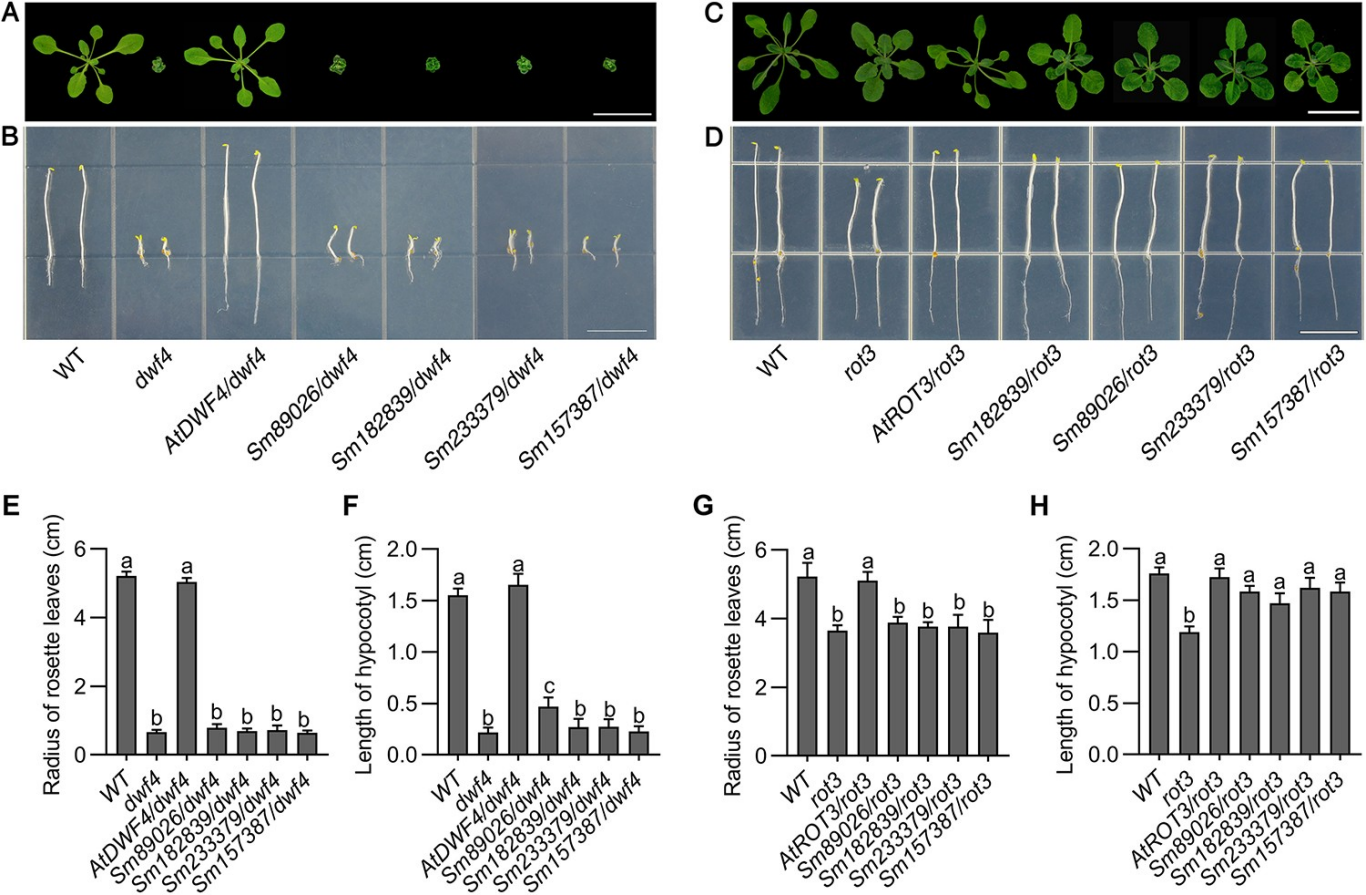
Phenotypes of WT and *SmCYP90* transgenic lines in *dwf4* and *rot3* mutants. (A) Morphology of WT, *dwf4*, *AtDWF4/dwf4* and *SmCYP90* transgenic seedlings grown 30 DAG in the light. Scale bar, 3 cm. (B) Morphology of WT, *dwf4*, *AtDWF4/dwf4* and *SmCYP90/rot3* genes transgenic seedlings grown on 1/2 MS medium 5 DAG in dark. Scale bar, 1.0 cm. (C) Morphology of WT, *rot3*, *AtROT3/rot3* and *SmCYP90/rot3* genes transgenic seedlings grown 30 DAG in the light. Scale bar, 3 cm. (D) Morphology of WT, *rot3*, *AtROT3/rot3* and *SmCYP90/rot3* transgenic seedlings grown on 1/2 MS medium 5 DAG in dark. Scale bar, 1.0 cm. (E) Comparison of the rosette with of WT, *dwf4*, *AtDWF4/dwf4* and *SmCYP90/rot3* transgenic seedlings grown 30 DAG in the light. (F) Comparison of the hypocotyl length of WT, *dwf4*, *AtDWF4/dwf4* and *SmCYP90/rot3* transgenic seedlings grown 5 DAG in the dark. (G) Comparison of the rosette with of WT, *rot3*, *AtROT3/rot3* and *SmCYP90/rot3* transgenic seedlings grown 30 DAG in the light. (H) Comparison of the hypocotyl length of WT, *rot3*, *AtROT3/rot3* and *SmCYP90* genes transgenic seedlings grown 5 DAG in the dark.

Among the enzymes involved in BR biosynthesis in *Arabidopsis*, DWF4 catalyzes the rate-determining step [19], and DWF4 acts as a 22α-hydroxylase [20]. The transgenic lines of *SmCYP90* genes in *dwf4* had smaller seedlings and shorter hypocotyl than WT (Fig 7A and 7B), indicating that *SmCYP90* genes do not encode enzymes with an equivalent function to that of (*At*)*DWF4*. The *DWF4* gene has been shown to encode a cytochrome P450 enzyme (CYP90B1) that only shares 43% amino acid sequence identity with CPD [21]. Since *Sm89026* could not rescue the *DWF4*, *Sm89026* is not a functional equivalent of *Arabidopsis DWF4* although Sm89026 was in a clade with *DWF4* rather than with *CPD* (Fig 3).

The polarized processes of cell elongation play a crucial role in morphogenesis of higher plants. The *ROT3* gene encodes a cytochrome P450 (*CYP90C1*) with domains homologous to the regions of steroid hydroxylases of animals and plants, confirmed that the *ROT3* gene controls polar elongation in leaf cells by an analysis of three *rot3* mutants obtained from different mutagenesis experiments [22]. The *rot3* mutants exhibit short petioles [23] (Fig 7C), and a small ratio of length to width than that of the WT. No transgenic plants of *SmCYP90* genes rescued the phenotypes of *rot3* as being shown in the seedlings grown for 4 weeks (Fig 7C).

## Discussion

As a class of essential plants hormones, BRs play key roles in regulating a broad aspect of plant growth and development. BRs are biosynthesized from campesterol via a 5-alpha reductase and several cytochrome P450 (P450) catalyzed oxidative reactions [24]. The BR biosynthetic and signaling pathways have been well characterized in *Arabidopsis* and other angiosperms, but our knowledge of these pathways is limited in other plant groups. Previously, it has been reported that the Lycophyte *S*. *moellendorffii*, an ancestral vascular plant, has physiological responses to the BRs and to the BR biosynthetic inhibitor, PCZ (propiconazole). This suggests that BRs are biosynthesized in *Selaginella*. Unfortunately, most BR intermediates found in *Arabidopsis* and rice were not detectable or only present at very low levels. So far, we do not know the biosynthetic process of BRs in *Selaginella*.

Based on ectopic expression and phenotypic complementation of BR biosynthetic mutants of *Arabidopsis*, we have studied the function of *DET2* and *CYP90* genes in non-seed plants using *SmDET2*, *Sm89026*, *Sm182839*, *Sm233379* and *Sm157387* isolated from *S*. *moellendorfii*. The results show that *Sm89026* (*SmCPD*) belongs to a clade with *CYP90A1* (*CPD*) and *CYP90B1* (*DWF4*) while *Sm182839*, *Sm233379* and *Sm157387* forms a distinct clade with *CYP90C1* (*ROT3*) and *CYP90D1* (Fig 3). *SmDET2*, *SmCPD* and *Sm1573872* are highly expressed in both leaves and strobili while *Sm233379* is only highly expressed in the leaves but not in the strobili of *S*. *moellendorfii* (Fig 4), implying their differential functions. We show that only *SmDET2* and *SmCPD* completely rescue *det2* and *cpd* mutant phenotypes, respectively (Figs 1 and 6), suggestive of their conserved BR biosynthetic functions. However, neither *SmCPD* rescues any other *cyp90* mutants, nor any other *SmCYP90* genes rescue any *cyp90* mutants. Yet, overexpression of *Sm233379* alters plant fertility and the expression of BR biosynthetic and metabolic genes, markers of BR functions (Fig 5). Taken together, *SmCPD* and *Sm233379* have a BR biosynthetic function. Furthermore, *SmCPD* is an equivalent of the *Arabidopsis CPD*, while *Sm233379* has no equivalent in *Arabidopsis* and the function of the other two *CYP90s* remains for future exploration.

The activation of BRs signaling pathway depends on a series of signal transduction components. But the most important receptor, BRI1, does not exist in *Selaginella*, suggesting that there are different signal pathways in *Selaginella* and *Arabidopsis*. However, most BR biosynthetic genes and signal components share high similarity in between *S*. *moellendorfii* and *Physcomitrella patens*, a primitive terrestrial non-vascular plant, but *P*. *patens* does not respond to brassinolide [25]. We infer that there is not conventional BR signal receptor to activate downstream transcription, although a relatively complete BR synthesis pathway exists, in *Selaginella*. This leads us to believe that castasterone precursor, the product of CPD, played a role as physiological active substance but not a hormone, as the responsive concentration is much higher in *Selaginella* than in *Arabidopsis* [8]. Another possibility is that there may be a kind of completely unknown BR receptors in *S*. *moellendorfii* and *P*. *patens*, having a signaling pathway significantly distinguished from that of *Arabidopsis*.

**Fig 8:**
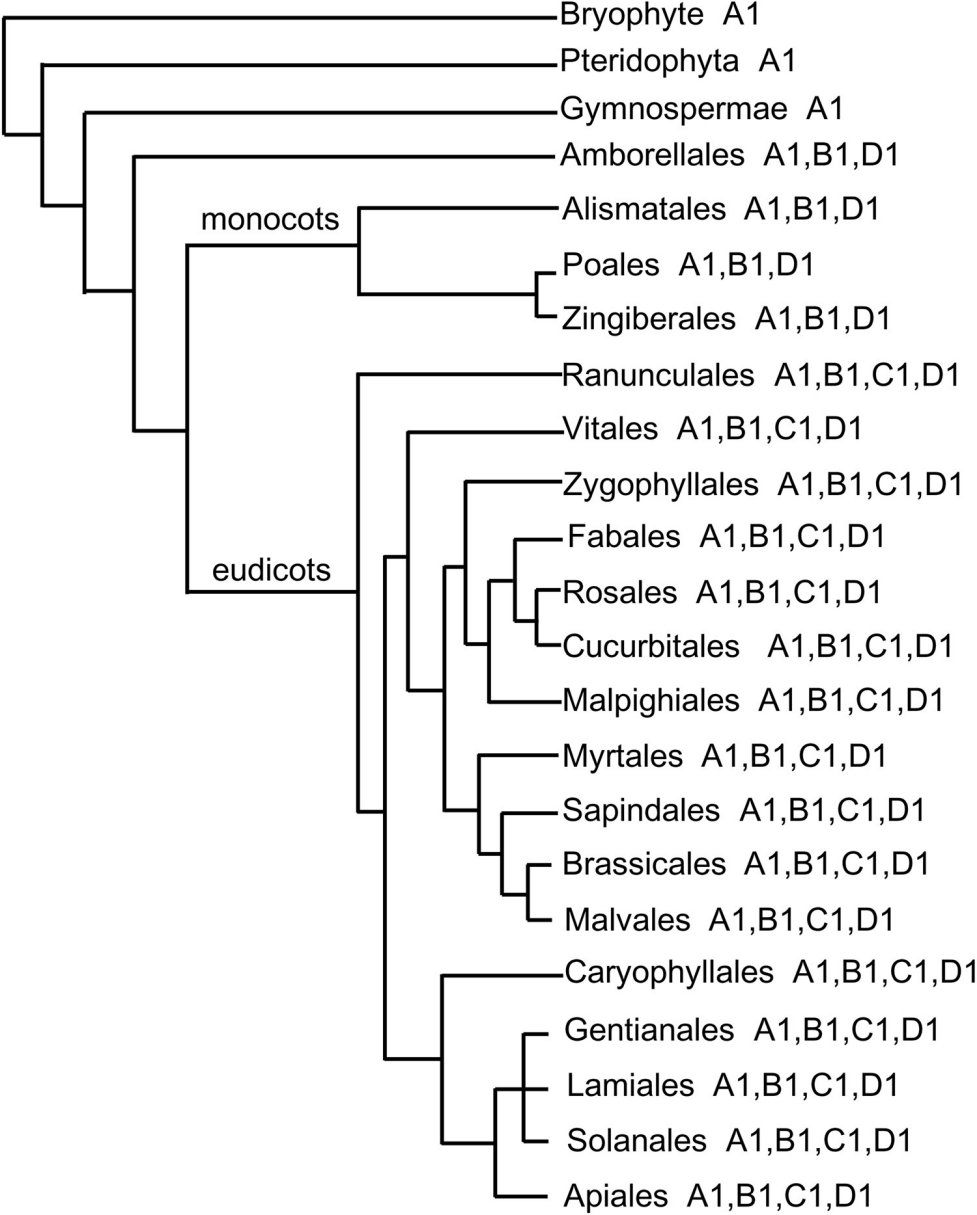
Distribution of CYP90 genes based on APG IV. A1, B1, C1 and D1 are short for *CYP90A1*, *CYP90B1*, *CYP90C1* and *CYP90D1*, respectively. *CYP90C1* is only discovered in eudicots.

The *CPD* encodes an enzyme having function in a key rate-limiting step, and *cpd* mutant shows extremely dwarf phenotypes with reduced fertility similar to the mutants of BR receptors, which is known as marker gene for estimating whether BR endogenous signal is strong or not according to CPD activity. Our results show that BR biosynthetic process in *Selaginella* is similar to that in *Arabidopsis* based on functional *SmDET2* and *SmCPD*, and the critical BR synthetic products existed in other primitive terrestrial plants [26]. These findings indicate that BR synthetic pathway has already appeared in early terrestrial plants before complete hormone-receptor signaling pathways arise (Fig 8). Together, our studies have been fruitful in identifying the function of putative genes involved in the biosynthesis of BR and analysis of differential expression, including *SmDET2* and *SmCYP90s*. This would lay the foundation for studying on the mechanism of BR function and understanding the origin of BR signal from primitive vascular plants. Biochemical approaches are likely to play increasingly critical role in filling the gaps of synthesis from the product of *SmDET2* to the substrates of *SmCPD* in future studies.

## Supporting information

S1 Fig Height of plants not significantly different from each other. Data are presented as the mean ±SD.
(TIF)

S2 Fig Radii of rosette leaves are not significantly different from each other. Data are presented as the mean ±SD.
(TIF)

S3 Fig Plants of *SmCYP90* overexpression were still sensitive to BRZ like as WT. Seedlings grown on 1/2MS medium 5 DAG in dark with/without 5μM BRZ. Scale bar, 1 cm.
(TIF)

S4 Fig Most of siliques from *Sm233379-OX* plants were abnormal. Scale bar, 2cm.
(TIF)

S1 Table Primers for gDNA PCR. (PDF)

S2 Table Primers for semi-quantitative RT-PCR. (PDF)

S3 Table The names of species for the construction of NL tree and gene ID. (PDF)
