# Supplementary figures and images for: Functional study of the brassinosteroid biosynthetic genes from Selagnella moellendorfii in Arabidopsis
Source: PLoS One. 2019 Jul 25;14(7):e0220038. doi: 10.1371/journal.pone.0220038 (PMC6658078; doi:10.1371/journal.pone.0220038)

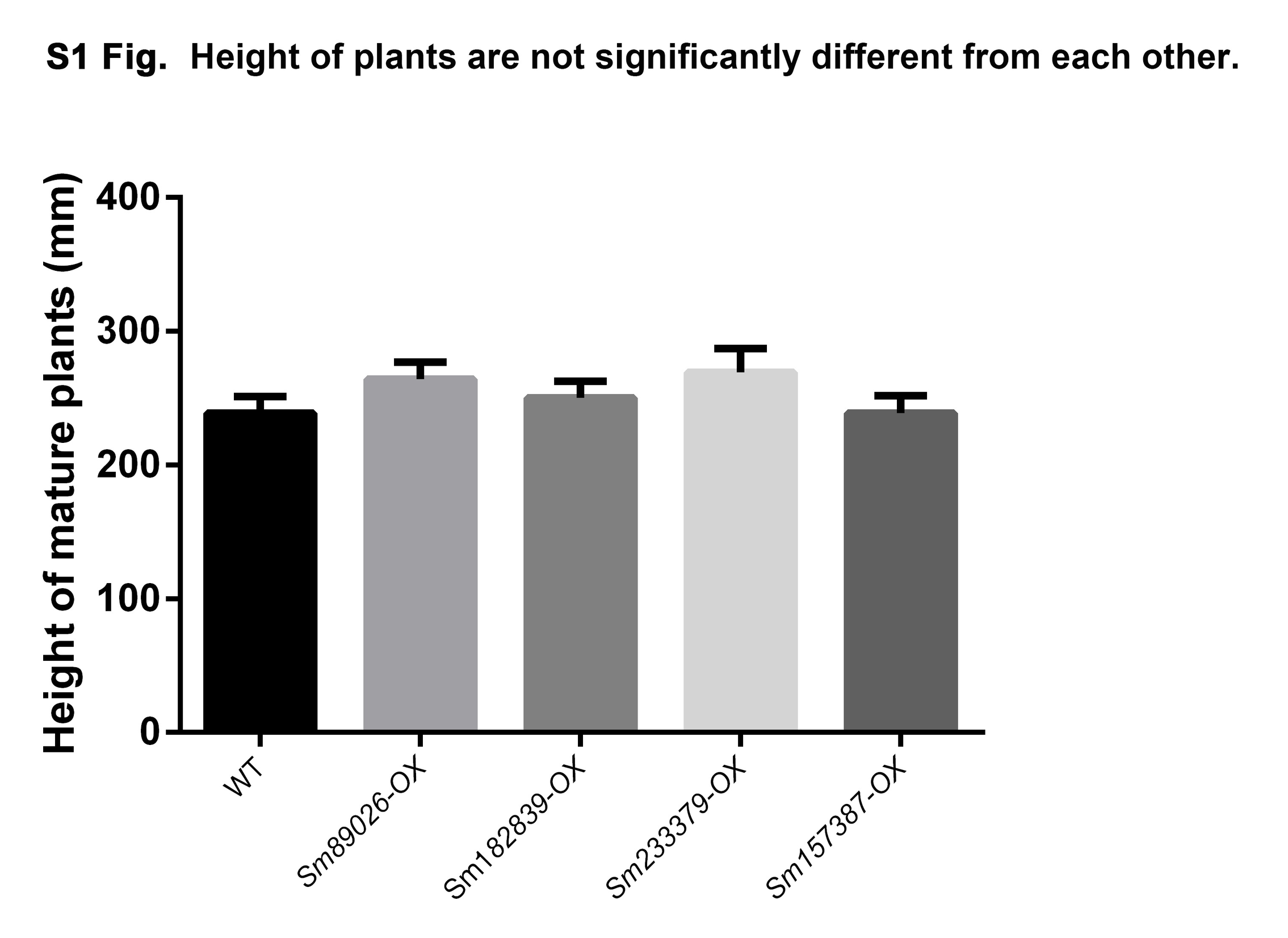

Supplement: S1 Fig — Data are presented as the mean ±SD. (TIF) [file pone.0220038.s001.tif]

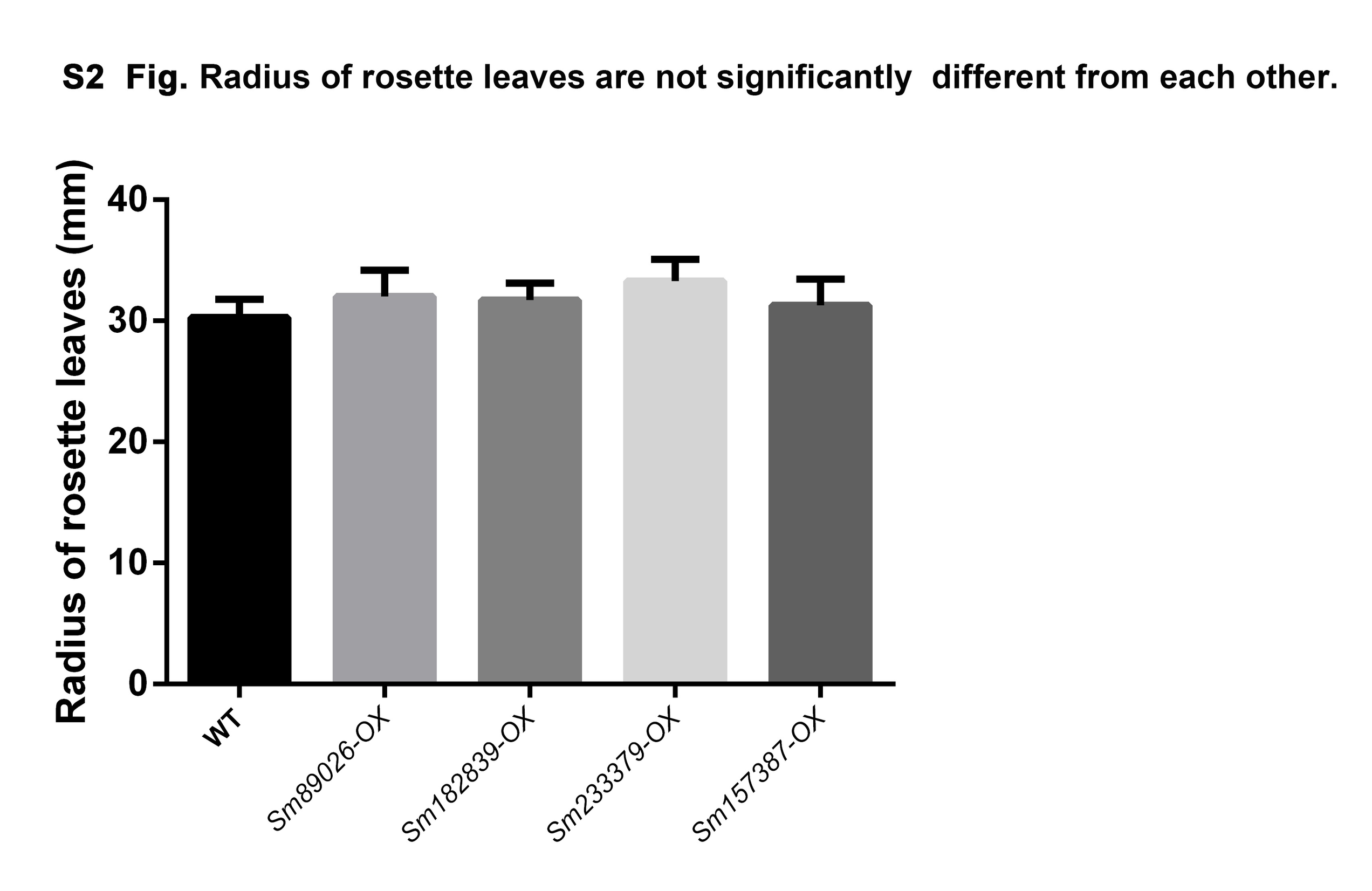

Supplement: S2 Fig — Data are presented as the mean ±SD. (TIF) [file pone.0220038.s002.tif]

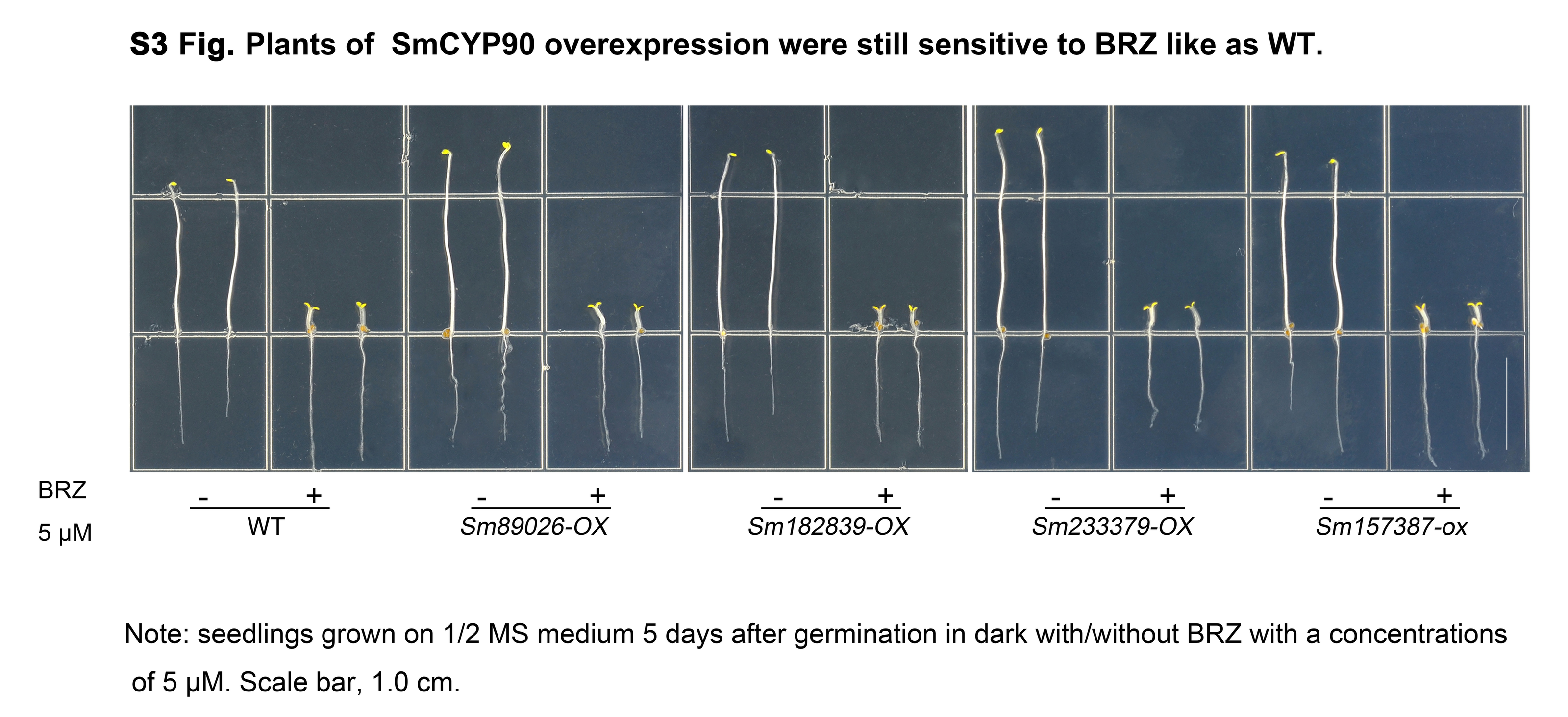

Supplement: S3 Fig — Seedlings grown on 1/2MS medium 5 DAG in dark with/without 5μM BRZ. Scale bar, 1 cm. (TIF) [file pone.0220038.s003.tif]

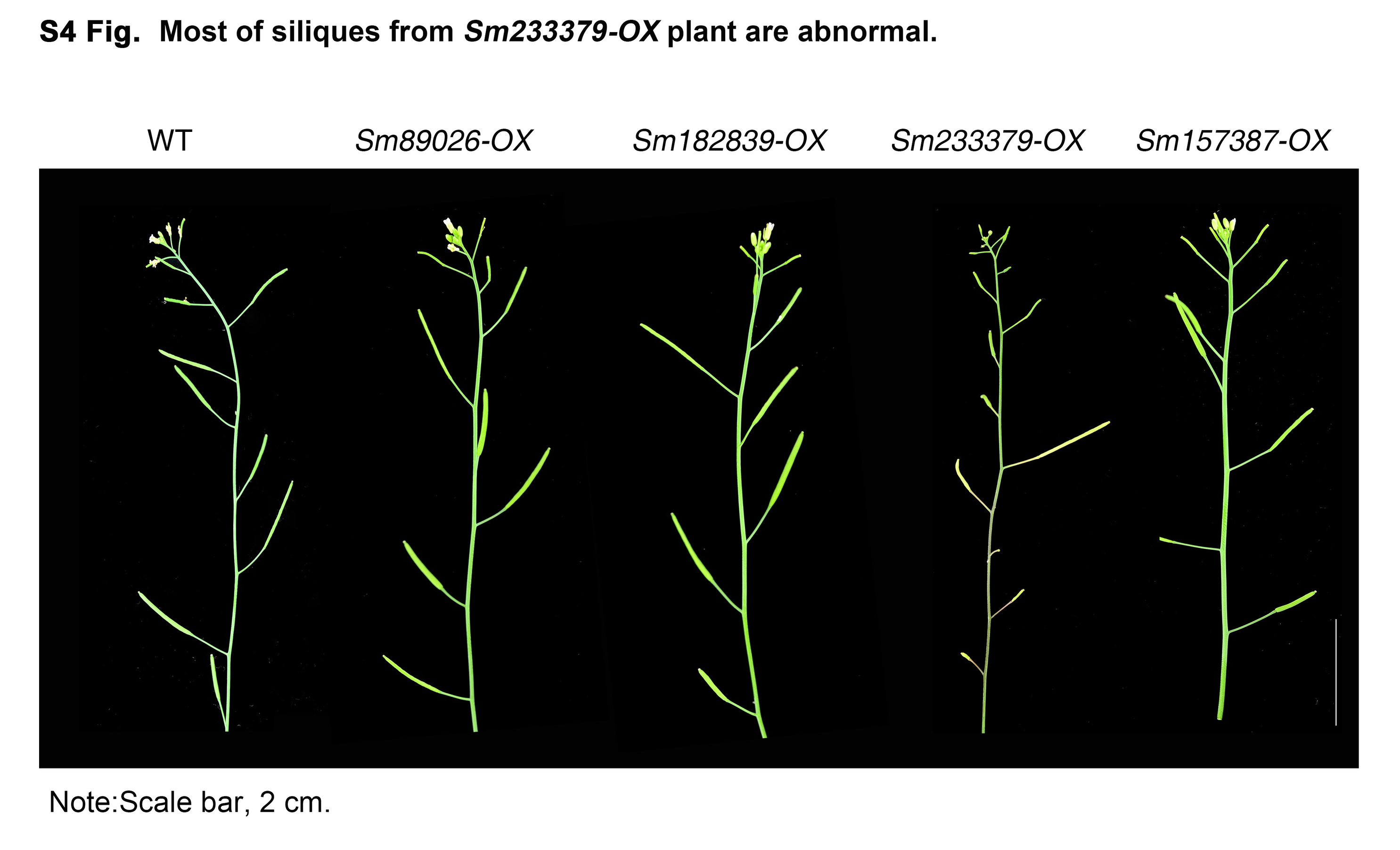

Supplement: S4 Fig — Scale bar, 2cm. (TIF) [file pone.0220038.s004.tif]
