# Supplementary material for: Functional study of the brassinosteroid biosynthetic genes from Selagnella moellendorfii in Arabidopsis
Source: PLoS One. 2019 Jul 25;14(7):e0220038. doi: 10.1371/journal.pone.0220038 (PMC6658078; doi:10.1371/journal.pone.0220038)
Supplement: S1 Table — (PDF) [file pone.0220038.s005.pdf]

S1 Table. Primers for gDNA PCR

Primer	Forward	Reverse
AtDET2	F:5'-ATTTCTTCCATTTTTCGG-3'	R:5'-AATTCCTCCAAGCTCCTT-3'
AtCPD	F:5'-AAGCGGTGAAGGCCATTGAAGAAGA-3'	R:5'-CGTGAAGCGCCGTGATTTTGCTACT-3'
AtDWF4	F:5'-TTCGAACATGGAGCTAGT-3'	R:5'-AGGGTTTCTCGTATTCTG-3'
AtROT3	F:5'-GGTT GCATACGTAAACAGTT-3'	R:5'-CGATCTCACTTGAAGATCAT-3'
SmDET2	F:5'-GTCGACGAGAACGAACGGGATCACTGC-3'	R:5'-GGTACCATGGAAGAAGAGCAGTGGTAC-3'
Sm89026	F:5'-GGTACCATGGAAGCACTGACCTTGAGC-3'	R:5'-GTCGACTAGAATCTGTGAGAGTTTGGTG-3'
Sm182839	F:5'-GGTACCATGGTGGATTCGAGAATTATG-3'	R:5'-GGATCCTGTAGTAACTCTAGAG ATGGAGAAG-3'
Sm233379	F:5'-GGTACCATGATTTCTTCTTCCACTGC-3'	R:5'-GTCGACGTTTA GATCATACAGCACAACTG-3'
Sm157387	F:5'-GGTACCATGGTGGATTCGAGAATTATG-3'	R:5'- GGATCCTA GATCGTGTAACACCACTGG-3'
