# Supplementary material for: Functional study of the brassinosteroid biosynthetic genes from Selagnella moellendorfii in Arabidopsis
Source: PLoS One. 2019 Jul 25;14(7):e0220038. doi: 10.1371/journal.pone.0220038 (PMC6658078; doi:10.1371/journal.pone.0220038)
Supplement: S2 Table — (PDF) [file pone.0220038.s006.pdf]

S2 Table. Primers for semi-quantitative RT-PCR

Primer	Forward	Reverse
AtCPD	F:5'-GTTCTTATCCTGCTTCCATTTG-3'	R:5'-AGCCACTCGTAGCGTCTCATT-3'
AtDWF4	F:5'-CGAAGGAAGGCTCTTTGAATG-3'	R:5'-CTTCAACGGCTTTAGGGCAA-3'
AtBAS1	F:5'-GTTCAGGACATTGTGGAGGAG-3'	R:5'-GGATAAAGCAACATAAGGACG-3'
AtACT2	F:5'-ACTCTCCCGCTATGTATGTCG-3'	R:5'-TGGACCTGCCTCATCATACTC-3' .
SmACT2	F:5'-TTACTCTTTCACCACCACCG-3'	R:5'-GCCAAAATAGAACCTCCGAT-3'
SmDET2	F:5'-CGAGAATCACAGCACCATAC-3'	R:5'-GCAAGGAAATAAGAACCGAG-3'
Sm89026	F:5'-TTGGGAGGGACGAGTTGTT-3'	R:5'-TATGGGCAGATGTTAGCACG-3'
Sm182839	F:5'-CAGAGAGGAAAACCTTGCG-3'	R:5'-CGTGTAACACCACTGGTAAAT-3'
Sm233379	F:5'-TCAAGAAGCATCTCACGC-3'	R:5'-TGTATTCATTCCAAGAAAGGC-3'
Sm157387	F:5'-ACCAGGGACTCCATACCACC-3'	R:5'-CTCCAAACGGCAGCACATAC-3'
