# Supplementary material for: Functional study of the brassinosteroid biosynthetic genes from Selagnella moellendorfii in Arabidopsis
Source: PLoS One. 2019 Jul 25;14(7):e0220038. doi: 10.1371/journal.pone.0220038 (PMC6658078; doi:10.1371/journal.pone.0220038)
Supplement: S3 Table — (PDF) [file pone.0220038.s007.pdf]

S3 Table. The name of species mentioned NL tree and gene ID

abbreviation	full name of species	gene ID
B1_Aco	*Aquilegia coerulea*	Aquca.010.00476
D1_Aco	*Aquilegia coerulea*	Aquca.037.00070
A1_Ath	*Arabidopsis thaliana*	AT5G05690
B1_Ath	*Arabidopsis thaliana*	AT3G50660
C1_Ath	*Arabidopsis thaliana*	AT4G36380.1
D1_Ath	*Arabidopsis thaliana*	AT3G13730.1
A1_Ccl	*Citrus clementina*	Ciclev10031576
B1_Ccl	*Citrus clementina*	Ciclev10025451m
C1_Ccl	*Citrus clementina*	Ciclev10025479m
D1_Ccl	*Citrus clementina*	Ciclev10025448m
A1_Cpa	*Carica papaya*	evm.model.supercontig_19_182
C1_Cpa	*Carica papaya*	evm.model.supercontig_6_188
B1_Cqu	*Chenopodium quinoa*	AUR62007979
B1_Csa	*Cucumis sativus*	Cucsa.201380.1
C1_Csa	*Cucumis sativus*	Cucsa.043240.1
D1_Csa	*Cucumis sativus*	Cucsa.360620.1
A1_Egr	*Eucalyptus grandis*	Eucgr.J00224
B1_Egr	*Eucalyptus grandis*	Eucgr.I02453.1
C1_Egr	*Eucalyptus grandis*	Eucgr.J02384.1
B1_Fve	*Fragaria vesca*	mrna14783.1
C1_Fve	*Fragaria vesca*	mrna14904.1
A1_Gma	*Glycine max*	Glyma.02G256800
B1_Gma	*Glycine max*	Glyma.02G057500.1
D1_Gma	*Glycine max*	Glyma.08G193900.1
A1_Gra	*Gossypium raimondii*	Gorai.010G198500
B1_Gra	*Gossypium raimondii*	Gorai.007G203700
C1_Gra	*Gossypium raimondii*	Gorai.003G012100.1
A1_Lus	*Linum usitatissimum*	Lus10014850
C1_Lus	*Linum usitatissimum*	Lus10028345
B1_Mac	*Musa acuminata*	GSMUA_Achr8T26330
A1_Mes	*Manihot esculenta*	Manes.07G128700
B1_Mes	*Manihot esculenta*	Manes.02G093100
C1_Mes	*Manihot esculenta*	Manes.02G099000
A1_Mgu	*Mimulus guttatus*	Migut.F00677
B1_Mgu	*Mimulus guttatus*	Migut.H00997
C1_Mgu	*Mimulus guttatus*	Migut.H02504.1
B1_Ppe	*Prunus persica*	Prupe.7G153500.1
C1_Ppe	*Prunus persica*	Prupe.7G162900.1
D1_Ppe	*Prunus persica*	Prupe.3G050900.1
A1_Ptr	*Populus trichocarpa*	Potri.010G189800
B1_Ptr	*Populus trichocarpa*	Potri.005G124000
C1_Ptr	*Populus trichocarpa*	Potri.007G018400.1
D1_Ptr	*Populus trichocarpa*	Potri.003G038200.1
B1_Rco	*Ricinus communis*	29634.m002158
C1_Rco	*Ricinus communis*	29634.m002059
A1_Sly	*Solanum lycopersicum*	Solyc06g051750
B1_Sly	*Solanum lycopersicum*	Solyc02g085360.2.1

C1_Sly	*Solanum lycopersicum*	Solyc02g084740.2.1
D1_Sly	*Solanum lycopersicum*	Solyc02g084740.2.1
Smo89026	*Selaginella moellendorfii*	Smo89026
Smo157387	*Selaginella moellendorfii*	Smo157387
Smo182839	*Selaginella moellendorfii*	Smo182839
Smo233379	*Selaginella moellendorfii*	Smo233379
B1_Tca	*Theobroma cacao*	Thecc1EG000402t2
C1_Tca	*Theobroma cacao*	Thecc1EG000287t1
B1_Vvi	*Vitis vinifera*	GSVIVT01018977001
C1_Vvi	*Vitis vinifera*	GSVIVT01018857001
D1_Vvi	*Vitis vinifera*	GSVIVT01016845001
AthCYP51	*Arabidopsis thaliana*	AT1G11680
